# A Call to Action: Now Is the Time to Screen Elderly and Treat Osteosarcopenia, a Position Paper of the Italian College of Academic Nutritionists MED/49 (ICAN-49)

**DOI:** 10.3390/nu12092662

**Published:** 2020-08-31

**Authors:** Tiziana Montalcini, Arturo Pujia, Lorenzo M. Donini, Lucia Frittitta, Fabio Galvano, Andrea Natali, Loris Pironi, Marisa Porrini, Patrizia Riso, Angela Albarosa Rivellese, Diego Russo, Giovanni Scapagnini, Mauro Serafini, Anna Tagliabue, Antonino De Lorenzo

**Affiliations:** 1 Department of Clinical and Experiment Medicine, University of Catanzaro Magna Grecia, Viale Europa, 88100 Catanzaro, Italy; pujia@unicz.it; 2 Department of Experimental Medicine, University of la Sapienza, Piazzale Aldo Moro 5, 00185 Rome, Italy; lorenzomaria.donini@uniroma1.it; 3 Department of Clinical and Experiment Medicine, University of Catania, Via Santa Sofia, 86-95123 Catania, Italy; lfritti@unict.it; 4 Department of Biomedical and Biotechnology Science, University of Catania, Via Santa Sofia, 86-95123 Catania, Italy; fgalvano@unict.it; 5 Department of Clinical and Experiment Medicine, University of Pisa, Lungarno Antonio Pacinotti, 43, 56126 Pisa, Italy; andrea.natali@med.unipi.it; 6 Department of Medical and Surgical Science, University of Bologna, Via Pupilli, 1, 40136 Bologna, Italy; Loris.pironi@unibo.it; 7 Department of Food, Nutrition and Environment Science, University of Milan, Via Festa del Perdono, 7, 20122 Milano, Italy; marisa.porrini@unimi.it (M.P.); patrizia.riso@unimi.it (P.R.); 8 Department of Clinical and Experiment Medicine, University of Naples Federico II, Corso Umberto I, 40, 80138 Napoli, Italy; rivelles@unina.it; 9 Department of Health Science, University of Catanzaro Magna Grecia, Viale Europa, 88100 Catanzaro, Italy; d.russo@unicz.it; 10 Department of Medicine and Health Science, University of Molise, Via F. De Sanctis, 86100 Campobasso, Italy; giovanni.scapagnini@unimol.it; 11 Department of Bioscience and food technology, University of Teramo, Via Renato Balzarini, 1, 64100 Teramo, Italy; mserafini@unite.it; 12 Department of Public Health, University of Pavia, Corso Str. Nuova, 65, 27100 Pavia, Italy; anna.tagliabue@unipv.it; 13 Biomedicine and Prevention, University of Tor Vergata, Via Montpellier, 1, 00133 Roma, Italy; delorenzo@uniroma2.it

**Keywords:** sarcopenia, muscle mass, handgrip strength, elderly, fractures, osteoporosis, chronic diseases, mortality, DXA, bioelectrical impedance analysis

## Abstract

Aging is a risk factor for the development of multiple chronic diseases, including cardiovascular disease, cancer and dementia. Life expectancy has increased in certain countries but this phenomenon is associated with a reduction of years of healthy life. Aging is associated with a number of physical and functional changes, especially sarcopenia. Sarcopenia is a clinical condition associated with a decrease in skeletal muscle and muscle strength, however, sarcopenia is a reversible condition. On the basis of the current scientific literature, sarcopenia could more appropriately capture an individual’s vulnerability to negative health-related outcomes since it represents an early form of the chronic diseases. Recognition of this clinical condition can improve the management of older individuals in many different clinical settings. Despite the limitations of the indirect methods used to study body composition, the Italian College of the Academic Nutritionists ME/49 recommends that health authorities and health professionals around the world should make a greater effort to diagnose sarcopenia earlier and to manage it more effectively. In line with the development of cancer screening, the use of two diagnostic tools for sarcopenia (BIA and DXA) should be implemented.

## 1. Introduction

The ageing of societies around the world and the increase in life expectancy in certain countries is unfortunately also associated with a demographic and epidemiological transition towards a frail population [1,2,3]. Frail older adults are vulnerable to stress, which increases the risk of adverse outcomes including chronic noncommunicable diseases (NCDs), disability and mortality [4].

Italy is a striking example of this phenomenon and is second to Spain as the country in Europe with the highest average life expectancy at birth [2], which increased by 2.8 years between 2000 and 2015 [3]. Although at an international level, this increase is seen as a public health success story; in approximately the same period of time, there has been an increase in several NCDs, which include a significant proportion of cases of cancer, dementia, falls and cardiovascular, metabolic and respiratory diseases [5].

Furthermore, the recent COVID-19 pandemic has shown peculiar epidemiological traits with high rates of hospitalization and mortality among older adults in Italy [6,7], as well as Black, Asian and minority ethnic populations in both the United Kingdom and the United States [8,9,10]. This confirms that elderly people in countries like Italy are as frail as poorer ethnic populations in other countries [8,9,10]. Therefore, the dramatic increases in life expectancy, attributable to improved medical care, in reality often correspond to a reduction of years of healthy life.

A longer healthy life should thus be the objective of all health policies. Consequently, there is a need for a new paradigm in the identification of the risk factors for chronic illnesses and the management of social and health behaviours throughout life.

A common condition in elderly individuals is a suboptimal diet, which is characterized by inadequate calorie and protein intake. Malnutrition affects approximately one-third of older people, particularly those who are resident in institutionalized facilities [11], where malnutrition may substantially influence the need for hospitalization as well as the levels of mortality in the short and medium term [12]. A suboptimal diet accounts for one in every five deaths globally, and nonoptimal intake of three dietary factors (whole grains, fruit, and sodium) accounts for more than 50% of deaths and 66% of disability attributable to diet [13].

Interestingly, it has been reported that many elderly patients/individuals experience a loss of muscle mass before a weight loss, which is often stable due to the fat mass increase [14]. When a reduced loss of muscle mass is associated with a reduced muscle strength, then an individual is affected by sarcopenia. Sarcopenia is the most prevalent syndrome among hospitalized elderly individuals/older medical inpatients [14,15] compared to clinical conditions such as malnutrition, frailty, and cachexia [14], and is also reported in community-dwelling older adults [16,17]. Sarcopenia is often associated with decreases in energy intake [18], however, there are many factors are responsible for the decline in muscle mass and muscle strength associated with aging. Among these, physical inactivity or a decreased physical activity level in aged individuals is a part of the underlying mechanisms of sarcopenia [19]. Consequently, sarcopenia could more appropriately capture an individual’s vulnerability to negative health-related outcomes [20]. Regardless of the causes, sarcopenia identifies elderly people at risk of reducing their years of healthy life and of developing NCDs since it represents a subclinical or early form of these chronic diseases.

Older adults with severe levels of sarcopenia are approximately two to five times as likely to have a disability as older adults with optimal normal muscle mass [21,22,23]. Despite a positive association bidirectional relationship between NCDs and disability, this association is largely ignored by the global health community [24]. Thus, both the prevention of NCDs and NCD-related disability are important elements of any proposed strategy for healthy aging [24].

This document focuses on the importance in identifying elderly individuals who are apparently disease free but are actually affected by sarcopenia, a condition associated with subclinical NCDs, which predicts incident adverse events and disability. Furthermore, this document highlights the urgent need to use inexpensive, noninvasive, quick and accurate methods, which are already available to diagnose sarcopenia and its different phenotypes. The overall aim is thus to improve the diet as well as all other treatments that can help prevent the consequences of sarcopenia in the elderly.

## 2. Sarcopenia and Its Different Phenotypes as Predictors of Adverse Outcomes

### 2.1. Definition and Assessment of Sarcopenia

Sarcopenia is a progressive, persistent and generalized skeletal muscle disorder that is associated with an increased likelihood of adverse consequences such as including falls, fractures, physical disability and mortality [25]. Expert groups worldwide have published several definitions of sarcopenia [26,27,28]. Some researchers suggested the term “age-related sarcopenia,” whereas others, the term “myopenia” to indicate the presence of clinically relevant muscle wasting. Despite the lack of consensus on an exact definition, the term “sarcopenia” has created awareness for this condition and its management. In its 2018 definition, the European Working Group on Sarcopenia in Older People (EWGSOP)2 uses low muscle strength (a measure of muscle function) as the principal parameter of sarcopenia, which is confirmed by the presence of low muscle quantity or quality [25]. Since detection of low physical performance predicts adverse outcomes, it is used to identify the severity of sarcopenia [25].

Muscle quantity can be assessed as total body skeletal muscle mass (SMM), as appendicular skeletal muscle mass (ASM), or as the muscle cross-sectional area of definite muscle groups or body locations [25]. This body compartment can be measured accurately using computed tomography (CT) or magnetic resonance imaging (MRI). However, the costs, availability and, for CT, radiation exposure, prevent their use in clinical practice. Compared with these techniques, dual-energy X-ray absorptiometry (DXA) is a less invasive and less costly method for quantifying muscle mass [29]. DXA could be considered as a reference standard (but not a gold standard) for measuring muscle lean body mass [30].

However, since bioelectrical impedance analysis (BIA) has been cross-validated in several populations for the study of the body composition, the use of BIA in the study of human body composition has grown rapidly in the nutritional field in the past two decades. BIA is a simple, noninvasive, reliable, repeatable, rapid and inexpensive method for estimating muscle mass by prediction equations [25,31]. However, to ensure reliability, several factors (such as hydration status food intake, and physical exercise) must be taken into account controlled [32,33,34,35].

### 2.2. Epidemiology of Sarcopenia and Its Phenotypes

Sarcopenia affects is prevalent in up to 15% of apparently healthy older adults [15,36], up to 80% of acutely hospitalized older patients [37] and up to 70% of older postacute rehabilitation inpatients [38]. Sarcopenia is significantly higher in individuals with several NCDs with the highest prevalence in individuals with type 2 diabetes mellitus (T2DM) and cardiovascular disease (CVD) (14.7–78.6% for T2DM and 15.5–44.7% for CVD) compared to individuals with dementia and respiratory disease (12.6–33.3% for dementia and 21–23.9% for respiratory diseases) [16].

Furthermore, the loss of muscle mass seems strictly associated with the loss of bone mineral density (BMD) [39]. Several studies support the high prevalence of sarcopenia in elderly patients with fragility fractures [40,41,42]. The loss of muscle mass in osteoporotic patients affected by osteoporosis is significantly more common than in subjects with normal BMD [39]. Consequently, the term “osteosarcopenia” has been coined to describe a subgroup of older persons affected by both osteoporosis (osteopenia/osteoporosis) and sarcopenia. Among community-dwelling populations, the prevalence of osteosarcopenia is up to 60% in both genders ≥75 years [43,44].

Furthermore, sarcopenia might worsen the effects of obesity in older adults, resulting in a particular phenotype called “sarcopenic obesity.” Sarcopenic obesity is a condition of reduced lean body mass in the context of associated with increased excess adiposity. However, to date, a unifying definition of this syndrome does not exist [45], thus, its prevalence varies widely between studies, depending on population characteristics and different definitions.

Different amounts of adipose tissue and muscle mass may alter the bone biology [43]. In most but not all studies, fat mass was an additional independent contributor to reduced BMD, especially in postmenopausal women [39,43], resulting in a particular phenotype called “osteosarcopenic obesity.” The negative link between total fat mass and whole-body BMD may reflect the increased bone resorption associated with the synthesis of inflammatory cytokines in visceral fat [39], but only in women, whereas in men, FM and BMD are not associated, probably due to the different hormonal as well as physical activity pattern [39]. However, again, a unifying definition of this phenotype as well as its prevalence data does not exist.

### 2.3. Sarcopenia-Related Outcomes

While age-related sarcopenia is considered to be a primary sarcopenia, a number of disease states and metabolic conditions (such as hypercatabolism and cachexia) can lead to secondary sarcopenia [25]. Sarcopenia has thus been associated with an increased risk of hospitalization, it also requires long-term care and doubles the risk of dying over a period of 3–10 years [46,47,48,49].

Sarcopenia is associated with cardiovascular disease (CVD) [16,50], respiratory disease [16] and cognitive impairment [16,51]. In addition, sarcopenia is associated with greater cardiovascular mortality and all-cause mortality [20]. However, sarcopenic obese older men were found to have a higher risk of all-cause mortality than obese and sarcopenic individuals, suggesting a negative synergic role for the increased fat mass and reduced muscle mass in explaining the relationship between sarcopenic obesity and mortality [20,52]. It has also been reported that sarcopenic obesity is a key cause of long-term disability in older adults [53,54,55,56,57].

Osteopenia and osteoporosis have also been associated with an increased risk of mortality irrespectively of fragility fractures [58,59,60]. However, osteosarcopenia leads to significantly worsened outcomes than seen in either condition alone. A recent meta-analysis found that the odds of a fracture in those suffering from sarcopenia was approximately twice as high as a nonsarcopenic older person [61]. Osteosarcopenia is also associated with significantly increased mortality (1-year mortality rate of 15.1% in osteosarcopenic patients vs. 5.1% in osteoporotic and 10.3% in sarcopenic patients alone [62]. This implies a greater risk of frailty [63] and institutionalization as well as significant socioeconomic costs.

In the context of the risk of communicable disease (CDs), sarcopenia predicts the risk of infection after surgery, and among hospitalized patients, sarcopenia is associated with a twofold increase in the risk of developing nosocomial infections [64,65]. Sarcopenia also predicts the risk for community-acquired pneumonia in the elderly [66,67].

The burden of outcomes related to sarcopenia rests at both the individual and societal levels. In Europe, there will be an estimated 72% increase in the number of sarcopenic patients by 2045 [64]. Sarcopenia and its various phenotypes will thus become a public health concern in the future.

## 3. Potential Mechanisms Linking Sarcopenia to NCDs and Mortality

**Figure 1:**
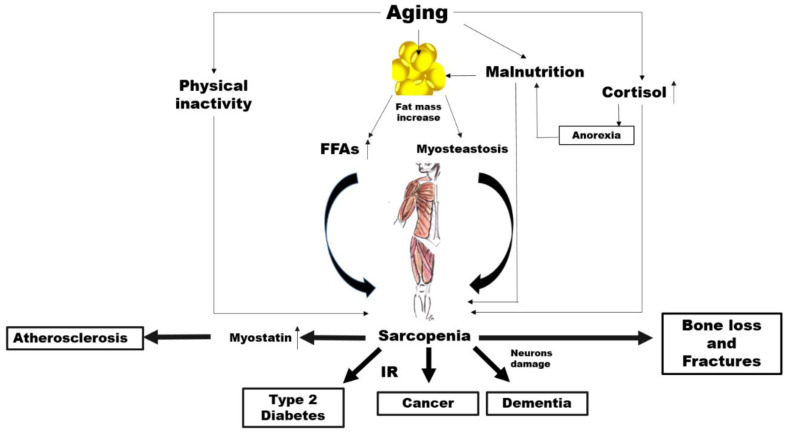
Potential mechanisms linking aging, sarcopenia and noncommunicable diseases (NCDs). FFAs: free fatty acids, IR: insulin resistance..

There is abundant evidence of the key crucial role of abnormal altered muscle metabolism in the genesis, and consequently prevention, of many common NCDs (Figure 1).

Aging is characterized by an increase in the percentage of body fat which generally translates to is associated with an elevated availability concentration of free fatty acids (FFAs) leading, in turn, to insulin resistance (IR) [68,69].

However, increased accumulation of fat around and within organs that normally contain only small amounts of fat, such as in skeletal muscle, can impair the normal physiological function of those organs [70]. This phenomenon, known as myosteatosis, occurs before the onset of strength and functional abnormalities as well as before the metabolic alterations such as IR and T2D in the elderly [71,72]. Among older adults, myosteatosis is related to decreased muscle strength [73].

Irrespectively of all these mechanisms, a substudy carried out within the Look AHEAD clinical trial demonstrated that individuals with diabetes have a greater fat mass and smaller lean mass regardless of ethnicity compared to controls [74]. Individuals with hyperglycaemia or diabetes have less appendicular lean body mass and a lower muscle quality, which possibly reduces the surface area for glucose transport [75]. On the other hand, a higher percentage of total lean body mass has been associated with a lower likelihood of current diabetes, prediabetes and insulin resistance in cross-sectional studies [76]. As skeletal muscle is responsible for the majority of the body’s postprandial glucose disposal, IR in this body compartment results in substantial whole-body metabolic disorders.

Of course, it is possible that changes in lean body mass represent both a risk factor and a consequence of impaired glucose states, irrespectively of changes in total body fat. In addition, physical inactivity, which is a common feature of ageing, is associated with a decline in mitochondrial oxidative function in the muscle [77], which involves a reduced capacity to oxidize fatty acids leading, again, to IR [77]. It is well known that IR characterizes the metabolic syndrome, which is a condition preceding frank diabetes [78]. There is also a strong correlation between insulin resistance and the risk of developing CVD [79]. Furthermore, IR seems to be closely associated with the increased incidence of and/or mortality from a broad range of cancers such as breast [80], colorectal [81], pancreatic [82] and liver [83] cancer. A first mechanism linking sarcopenia to NCDs might thus involve IR.

The mechanical force applied to the bone, which is critical to bone health, could clarify another possible link between sarcopenia and NCDs. It is attributable to come from two primary sources: external gravitational loading (via ground reaction forces) and internal loading (via muscle contractions). These forces are reduced significantly with a sedentary lifestyle as in advanced age. Several researchers have postulated whether the loss of one tissue with ageing might predispose the loss of the other. Exposure to microgravity with spaceflight represents a valid model to understand the role of muscle on bone metabolism.

The accelerated loss of bone and muscle mass as a result of microgravity is well documented over the decades [84]. Interestingly, under this condition, muscle atrophy precedes the decline in bone mass [85]. In fact, muscle is a source of particular molecules, namely, myokines, which stimulate bone formation and also contribute to bone loss [86,87]. Most interestingly, in spinal cord injury (SCI), which is a clinical condition associated with both sarcopenia and osteoporosis, the primary cause of bone loss is the removal of muscle-induced mechanical stimuli on the bone [88]. The alteration in the skeletal muscle metabolism associated with a reduced mechanical loading might thus play a role in the association between sarcopenia and common NCDs, such as osteoporosis.

Other possible links specifically concern cardiovascular diseases. Myostatin is a member of the transforming growth factor-β (TGF-β) super family, which is predominantly produced in skeletal muscle in response to diverse stimuli, including oxidative stress, inflammation and others [89].

Myostatin increases in sarcopenia, by driving ubiquitin-proteasome-mediated protein degradation [90,91], while a myostatin deficiency has been shown to have beneficial effects on muscle size and mass [92], as well as adiposity and insulin sensitivity [93]. Myostatin has also been proposed as a putative new mediator of progressive atherosclerotic lesions, heart dysfunction, cardiac fibrosis, and cardiovascular cachexia [94,95].

The risk of neurodegenerative diseases may be another factor in the link between sarcopenia and NCDs. It has been reported that cortisol concentrations increase with age [96]. While ageing, the increase in cortisol levels can have various effects on body composition and multiple systems in older people [97]. The activity of 11β-hydroxysteroid dehydrogenase type 1, an enzyme which catalyses the synthesis of cortisol, is enhanced in various tissues, such as the central nervous system, skeletal muscles and bones [98]. As a catabolic hormone, higher cortisol levels are linked with weight loss, muscle mass reduction, bone loss as well as reduced physical performance and anorexia, leading to the fundamental characteristics of frailty [96,99,100]. High cortisol levels make neurons more vulnerable to several kinds of insults, and Alzheimer disease (AD) patients have mildly increased levels of cortisol compared to controls [101,102].

## 4. Now Is the Time to Screen for Sarcopenia

On the basis of all these data, a screening strategy is urgently needed that detects sarcopenia in its early stages. Importantly, the average cost of health care related to an individual affected by sarcopenia is three times higher than a nonsarcopenic individual [103].

Several factors need to be assessed when making a decision about disease screening, including how common, fatal or complicated by serious health impacts the condition to be screened is, and also whether treatment is available. Of course, screening programs are tailored toward those individuals who the disease is most likely to affect and where the most benefit can be gained. The benefit-to-harm ratio of screening for sarcopenia increases as an individual ages due to the increase in the prevalence of sarcopenia. We therefore believe that screening for sarcopenia, as with other types of screening, should be planned and promoted by health authorities and confined to a specific section of the population based on certain risk factors (i.e., elderly individuals).

An ideal screening test should be noninvasive, cost effective, easily performed and of course accurate. However, with reference to MRI, CT, DXA and BIA, several publications have concluded that currently, there are no practical diagnostic methods for sarcopenia [104,105,106]. Several proponents of screening tools have thus tried to exploit screening questionnaires, diagnostic grids, or prediction equations [107,108,109,110,111]. However, these screening tools provided contradictory results in many cases due to their scarce sensitivity but high specificity (in other words, only severe cases may be detected with a high rate of false negatives). Nevertheless, their predictive values in diagnosing sarcopenia are generally acceptable, especially for primary prevention [112,113]. Unfortunately, there is relatively limited evidence from experimental studies of their effectiveness and it is unclear which of these current tools is the most effective in screening for sarcopenia in the community. While these tools might be useful in primary prevention, in the secondary prevention of individuals who already have sarcopenia, these tools cannot be used for early diagnosis. On the other hand, BIA and DXA could have a primary role as they have most of the characteristics of an ideal tool.

In line with the development of cancer screening, especially for breast cancer, the use of these diagnostic tools for sarcopenia could be improved by addressing some of the uncertainties that some physicians have highlighted [114].

Briefly, breast cancer screening is the regular examination of asymptomatic, apparently healthy women. The aim is to identify breast cancer as early as possible, so that women can receive timely and effective treatment in order to prevent complications and mortality. In this sense, the screening for sarcopenia could be similar to that of breast cancer. Most national scientific groups recommend mammograms from the age of 40, however, international policies differ with respect to the target age group to be screened. In any case generally there is consensus on the use of mammography [114]. A mammogram exposes a woman to 0.4 mSv, which is approximately equivalent to the amount of radiation a person would expect to get from natural background exposure over 7 weeks. On the other hand, one chest X-ray exposes the patient to about 0.1 mSv [115], which is about the same amount of radiation people are exposed to naturally over the course of about 10 days [115]. Below 10 mSv, no direct epidemiological data support an increased cancer risk. However, this does not mean that this risk is not present. Theoretical concerns about radiation-induced breast cancer from exposure to repeated mammography have been raised, however, the potential benefits are thought to outweigh the risks. For example, the benefit-to-harm ratio is estimated to be 48.5 lives saved per 1 life lost due to radiation exposure [116]. There are no doubts, therefore, about the importance of this screening programme.

Yet curiously, there seems to be concern in relation to the radiation dose from DXA, if used for the screening of sarcopenia [105,106]. The potential risk for an individual associated with DXA techniques used for the assessment of body composition is very small because the radiation doses are low. Effective doses for whole-body DXA examinations were found to range between 0.0052 and 0.008 mSv [116], thus approximately 200 times lower than that from mammography [115]. It is clear that DXA is considerably safer than mammography. It is estimated that one in two women will have at least one false-positive mammogram result [117] and false positive results can provoke anxiety and increase costs [118].

Wide ranges were noted for the percentage of mammograms referred as judged to be atypical abnormal (1.2–15.0%), as well as for the positive predicted value (PPV) (3.4–48.7%), which represent the probability of presenting a breast cancer in the case of a positive screening test [119]. However, mammography is viewed as the most accurate technique for screening purposes. So, how accurate are the current instruments used for the body composition assessment?

There seems to be a strong correlation between hydrodensitometry (the reference method for the assessment of body composition in the past) and DXA lean mass assessment (correlation coefficients > 0.86) with only small differences in the mean body fat compartment levels [120,121], which enabled DXA technology to be validated. The accuracy of body-composition measurements in vivo by DXA was also assessed in pigs (because their body weights and compositions are close to those of human neonates) [122]. As coefficients of variation (CVs), a precision of <2%, <3% and 2% for fat mass, lean mass and percentage fat, respectively, has been reported [123]. However, CT and MRI, which are particularly accurate in assessing muscle and fat areas in cadaveric studies, are still considered as reference methods. Several studies have demonstrated that appendicular lean soft tissue mass measured by DXA is highly correlated with both MRI and CT measurements of skeletal muscle volume [124,125,126]. Although DXA tends to underestimate the extent of sarcopenia, overall, there is a good correlation between the measurements of skeletal muscle mass in the lower limbs using DXA and those using MRI and CT [126,127]. There is also a good agreement between DXA and CT for abdominal total tissue mass and fat mass [128]. MRI and CT are expensive, time consuming and, in the case of CT, requires excessive radiation doses and are thus impractical in clinical settings. Consequently, DXA is now preferred to assess body composition.

A study comparing the performance of five screening tools for the risk of sarcopenia, reported a PPV for the algorithm of the EWGSOP (in which the skeletal muscle index, SMI, was assessed using DXA) from 17.5% to 42.5%, with a sensitivity range of 33.3–70.6% and a specificity range of 88.5–91.1% [129]. These values are very similar to that of mammography.

One source of error in the assessment estimation of body compartments composition by DXA is the hydration status of the subject. However, it has been reported that the magnitude of error in fat or fat-free mass would not exceed 0.5 kg, even under conditions of extreme physiological variance in hydration status [130].

Bone mineral density (BMD) assessed by DXA is highly correlated with bone histomorphometry (r > 0.90) in patients with a number of metabolic bone diseases [131,132,133], as well as undergoing hip replacement [134]. Using the WHO cut-off values for the definition of osteoporosis, DXA can detect up to 88.2% of possible cases of osteoporosis (sensitivity) with a specificity of approximately 63% and a PPV of more than 80% [135], which is much higher than that of mammography. Typical in vivo BMD precision values reported for humans are 1–2% with a high accuracy (4–10%) [136]. Although there are differences in BMD values among the scanners, due to different calibration methods and different edge detection methods, nevertheless BMD values obtained on different machines were highly correlated (r > 0.99). BMD measurements do not fully account for changes in trabecular architecture, tissue properties and accumulation of microdamage [137,138]. In any case, BMD measurements are currently the standard method for diagnosing osteoporosis.

How can the overall performance of a screening tool be improved? Most studies have reported an increase in sensitivity by adding breast ultrasound to the mammogram. The PPV for ultrasound alone ranges from 6% to 11%, but is as high as 56% when combining mammography with ultrasound, which gives a sensitivity of approximately 80% [139,140,141,142,143,144,145,146]. However, hand-held breast ultrasound screening is operator dependent [147]. Other limitations in implementing widespread screening ultrasound include a lack of standardized scanning protocols [147].

In order to study body composition, one solution would be to combine a DXA scan with a BIA examination. In fact, both methods can be used to measure lean mass [25]. The advantages of DXA (precision, accuracy and assessment of BMD) fit well with those of BIA (noninvasiveness and low cost) jointly contributing to the diagnosis of sarcopenia, osteosarcopenia and sarcopenic obesity. A BIA can predict a reduced ASMM (with DXA as the gold standard) with a PPV of 73%, a sensitivity of 80% and a specificity of 91% in geriatric patients [148], better than breast ultrasonography alone and similar to the combination of mammography plus ultrasound. A recent study showed that compared to DXA, BIA is highly reliable in terms of assessing appendicular lean mass, with an intraclass correlation coefficient (ICC) of 0.89 (95% CI: 0.86–0.92), when performed by the same operator, and an ICC of 0.77 (95% CI: 0.72–0.82), when performed by two different operators [149]. All the potential systematic bias in the estimation of lean body mass measurements by BIA would be overcome by combining BIA with a more precise technique such as DXA.

In Italy, the average cost of breast cancer screening is around 95 euros, whereas sarcopenia screening would only cost around 76 euros [150].

## 5. Recommendations

It is time that health professionals made a greater effort to diagnose sarcopenia earlier and to manage it more effectively. Based on the available evidence supporting that sarcopenia as predictive biomarker of several NCDs and mortality, the Italian College of the Academic Nutritionists ME/49 (ICAN-49) recommends the following:

1. Nutritionists are encouraged to screen for sarcopenia using a combination of DXA plus BIA as screening tools for secondary prevention.
2. General practitioners (GPs) as well as other health care professionals should suggest a screening for sarcopenia in elderly individuals.
3. Nutritionists as well as other health care professionals (as geriatrics, internists, neurologists and cardiologists) should ask National health authorities to plan screening for the early diagnosis of sarcopenia and its various phenotypes in the elderly.
4. Nutritionists are encouraged to ask National health authorities for provide DXA and BIA equipment to the nutritional units and to reimburse the costs of all the treatments for those diagnosed with sarcopenia including where it is a secondary condition to chronic diseases.
5. Nutritionists should collaborate with health care policy makers and health care providers regarding medical claims and common standards of screening technologies.

Specifically, we propose that adults older than 65 year undergo a measure of handgrip strength (prescreening), which is known to have a high specificity for identifying those at risk of dynapenia. Dynapenia, the age-associated loss of muscle strength, represents a clinical marker of mobility loss and deficiency in instrumental activities of daily living, better than the decline in muscle mass [151]. The handgrip strength test makes easier the strength measurement, and it is inexpensive. However, individuals who lose loss strength in addition to muscle mass are more predisposed to the loss of mobility than those who only lost muscle strength. Thus, in order to rule out the presence of sarcopenia and better dissect the contribution of the bone loss from muscle loss in the reduction of ASMM, the next step (I level exam) could be to perform a DXA scans in those who are positive for low grip strength (whole body as well as femoral and spinal scan). Since the evaluation of lean mass by DXA might underestimate the age-related decrease in muscle mass, due to the increase in total body water with ageing, a second step (II level exam) could be to perform BIA in order to confirm the reduction in ASMM.

To improve knowledge on this theme, ICAN-49 recommends the following:

1. Promotion of centralized data collections in epidemiological studies on older adults.
2. Development of RCTs on new treatments for sarcopenia in individuals with either primary or secondary sarcopenia.
3. GPs should receive continuing medical training regarding sarcopenia.
4. The government should fund information and prevention campaigns, in collaboration with patient associations and scientific research groups.

The aim of screening for sarcopenia is to counteract all the negative complications derived from sarcopenia i.e., falls, fractures, loss of independent living and, ultimately, NCDs and mortality. Since the aetiology of sarcopenia in the elderly is multifactorial, there need to be different treatment approaches. The scope of this document does not include a review of all the studies on the dietary and nondietary approaches to treat sarcopenia. However, here, we briefly summarize the cornerstones of sarcopenia treatment.

Although in some publications, the quality of evidence is not high, a number of systematic reviews and meta-analyses have shown several positive effects of nutritional and exercise interventions for treating sarcopenia in older people [152,153,154,155,156,157,158,159,160,161,162,163].

Skeletal muscle (SM) makes up approximately 40% of total body weight, consisting of 50–75% of total proteins. Inadequate dietary protein intake alters the whole-body protein balance inducing muscle catabolism to provide amino acids to allow for protein synthesis [164]. Consequently, there is a loss of muscle mass and bone, which then influences hormone synthesis, immune system response (and thus, susceptibility to infections) and autonomy [164]. A protein intake of between 1.2 and 2.0 g∙kg^−1^∙day^−1^ or higher for elderly adults has been recommended [165,166]. However, elderly adults typically eat less than younger adults do, and a lower protein intake has been reported [166,167]. It has been estimated that approximately 40% of adults of both genders have dietary protein intakes that are below the recommended dietary allowance (RDA) [167,168]. A recent study on the elderly in Italy and Brazil reported that the diet did not provide an adequate amount of protein to maintain functional status in older adults (only ~1.0 g∙kg^−1^∙day^−1^) [169], thus supporting the need to increase the protein intake for the elderly [170]. However, even with an appropriate supply of amino acids, reduced energy intake causes a reduction in protein synthesis with a consequent reduction in the size of myofibrils [171]. These findings indicate that to maintain muscle mass, an individual’s energy intake is crucial.

A higher intake of animal protein than plant protein was significantly associated with a 40% difference in the loss of lean body mass over time in elderly older adults, in favour of animal protein [172]. Notably, the ingestion of animal proteins (i.e., from milk) compared with the ingestion of vegetal proteins seems to stimulate muscle protein synthesis to a greater extent after resistance exercise [170,173,174]. Greater amounts of plant-based proteins are necessary to stimulate the anabolic mechanisms in the muscle mass than animal-based protein. The beneficial effects of the animal protein seem to be mainly attributable to the content of branched-chain amino acids (BCAAs) (especially leucine), which are potent stimulators of muscle protein synthesis via the activation of the mammalian target of rapamycin [175]. Indeed, lower systemic concentrations of BCAAs have been found in older adults/elderly individuals affected with sarcopenia than those that were nonsarcopenic [175].

A recent review [176], which summarized the results from 12 RCTs, concluded that the combination of whey protein with exercise improves muscle mass quantity, quality as well as physical performance [152,153,154,155,156,157,158,159,160,161,162,163,177]. However, it is difficult to establish from these studies what the exact contribution of whey is. Trials in older adults with whey supplementation alone have demonstrated that a daily dietary supplementation of 35 g of whey is likely to improve sarcopenic biomarkers in frail or sarcopenia individuals [163,178,179,180,181]. Although the greatest benefit for older sarcopenic individuals is resistance exercises [182], a meta-analysis concluded that dietary protein supplementation can be, if the usual protein intake is less than 1.6 g protein/kg/day, both sufficient and necessary to optimize resistance exercise training (RET)-induced gains in muscle mass and strength [183]. There appear to be no detrimental effects of increasing protein intake on bone health, [184]. It can thus be concluded that nutritional treatment plays a key role in the treatment of sarcopenia.

Although the number of publications is limited, there is also a growing literature pointing to the benefits of supplementing with high protein oral nutritional supplements enriched with β-hydroxy-β-methylbutyrate to address muscle-related problems that develop with aging and chronic disease [185]. Vitamin D and calcium supplementation also have a positive effect, especially among institutionalized older individuals with osteosarcopenia. Vitamin D and calcium supplementation help reduce the risk of hip fracture (by 16–33%) and any fracture (by 5–19%) [186]. Although vitamin D and calcium doses varied considerably among the studies, it is generally accepted that at least 800 IU/day should be added to a minimum of 500 mg/day of calcium supplementation (when an individual is unable to get adequate calcium through their diet) to have an effect on fracture risk [187]. Vitamin D supplementation of individuals presenting a 25(OH)D level less than 30 nmol/L also results in a significantly greater improvement in muscle strength, compared with those who present a 25(OH)D level of at least 30 nmol/L [188].

Several studies have also highlighted that RET is one of the most effective nonpharmacological strategies to increase BMD [189,190,191,192,193,194]. In fact, progressive resistance exercise is able to stimulate osteoblastogenesis [195].

Currently, there are no approved medications for treating sarcopenic obesity. Reviews seem to suggest that resistance exercise is essential in increasing muscle strength and physical performance parameters of people with sarcopenic obesity, whereas reducing calorie and fat intake positively affect body fat mass [196,197,198,199,200]. Overall, this evidence suggests that dietary interventions in older people should not focus only on increasing muscle strength, but also on optimizing muscle quality and improving muscle performance [72].

ICAN-49 recommends the following:

1. A protein intake of 1.2–1.5 g∙kg^−1^∙day^−1^ or higher for elderly adults, according to the nutrient intake, renal function and severity of sarcopenia, with at least 20–35 g/daily of whey protein, in conjunction with resistance exercise for a person with sarcopenia. Higher doses of protein (up to 2 g/day) may be appropriate in persons with severe illness or a catabolic status
2. Vitamin D, from dietary and supplemental sources, to any older person with vitamin D deficiency of insufficiency (at least 800 UI per day or more according to serum concentrations).
3. Calcium, from dietary and supplemental sources, should be in adequate amounts (i.e., at least 1200 mg per day).
4. Patients/older individuals at risk of or with established sarcopenia should be encouraged to be involved in regular physical activity.

These recommendations apply to both primary sarcopenia and secondary sarcopenia. In secondary sarcopenia, the diseases related to sarcopenia also need to be treated.

## 6. Conclusions

Despite the limitations of the indirect methods used to study body composition (particularly BIA and DXA) and the concerns related to standardizing protocols and treatments for sarcopenia, it is now strongly recommended that health authorities around the world should screen for sarcopenia. This treatable clinical condition has huge costs, precedes the appearance of diabetes, cancer, and dementia, and predicts mortality. Measures should be set up for the early identification of those individuals affected by sarcopenia and to treat them by combining nutritional interventions and exercise.

Cancer screening has several limitations, especially related to the ability of the technologies used to identify individuals who are really affected by cancer together with their invasiveness. However, these limitations have not prevented health authorities and the scientific community from implementing these screenings.

Accordingly, we strongly believe that nutritionists should start addressing this challenge and acquire diagnostic tools, rather than relying on alternative or surrogate tools. DXA has many advantages in terms of accuracy, simplicity, low cost, low radiation exposure, and low scanning time, and its role in sarcopenia diagnosis is thus emerging as the reference assessment technique in muscle mass evaluation. The assessment of body composition appears to be less reliant on underlying assumptions than most other methods. BIA is a popular, simple and portable technique for the estimation of body composition. It does not require skilled staff, is relatively inexpensive, and does not expose patients to radiation. Several studies have validated the use of BIA in determining appendicular muscle mass.

Treatment programs, including nutritional interventions associated with exercise training, may improve the quality and function of muscle mass. However, many older people have difficulties with swallowing, thus oral supplementations need to be prescribed.

If all the actions included in this document are implemented by nutritionists as well as other health care professionals, it should lead to a growing interest of national health authorities as well as the scientific community in this complex condition, and, as a consequence, a delay in the physical disability and mortality in older people.
